# Stimulation of the Subthalamic Nucleus Changes Cortical-Subcortical Blood Flow Patterns During Speech: A Positron Emission Tomography Study

**DOI:** 10.3389/fneur.2021.684596

**Published:** 2021-05-26

**Authors:** John J. Sidtis, Diana Van Lancker Sidtis, Vijay Dhawan, Michele Tagliati, David Eidelberg

**Affiliations:** ^1^Brain and Behavior Laboratory, Geriatrics Department, Nathan Kline Institute, Orangeburg, NY, United States; ^2^Department of Psychiatry, School of Medicine, New York University Langone, New York, NY, United States; ^3^Department of Communicative Disorders and Sciences, New York University Steinhardt School, New York, NY, United States; ^4^Center for Neurosciences, The Feinstein Institute for Medical Research, Manhasset, NY, United States; ^5^Department of Neurology, Cedars-Sinai Medical Center, Los Angeles, CA, United States

**Keywords:** Parkinson's disease, deep brain stimulation, Positron Emission Tomography, speech, subthalamic nucleus

## Abstract

**Background:** Deep brain stimulation of the subthalamic nucleus (STN-DBS) is an effective treatment for Parkinson's disease (PD) but can have an adverse effect on speech. In normal speakers and in those with spinocerebellar ataxia, an inverse relationship between regional cerebral blood flow (rCBF) in the left inferior frontal (IFG) region and the right caudate (CAU) is associated with speech rate. This pattern was examined to determine if it was present in PD, and if so, whether it was altered by STN-DBS.

**Methods:** Positron Emission Tomography (PET) measured rCBF during speech in individuals with PD not treated with STN-DBS (*n* = 7), and those treated with bilateral STN-DBS (*n* = 7). Previously reported results from non-PD control subjects (*n* = 16) were reported for comparison. The possible relationships between speech rate during scanning and data from the left and right IFG and CAU head regions were investigated using a step-wise multiple linear regression to identify brain regions that interacted to predict speech rate.

**Results:** The multiple linear regression analysis replicated previously reported predictive coefficients for speech rate involving the left IFG and right CAU regions. However, the relationships between these predictive coefficients and speech rates were abnormal in both PD groups. In PD who had not received STN-DBS, the right CAU coefficient decreased normally with increasing speech rate but the left IFG coefficient abnormally decreased. With STN-DBS, this pattern was partially normalized with the addition of a left IFG coefficient that increased with speech rate, as in normal controls, but the abnormal left IFG decreasing coefficient observed in PD remained. The magnitudes of both cortical predictive coefficients but not the CAU coefficient were exaggerated with STN-DBS.

**Conclusions:** STN-DBS partially corrects the abnormal relationships between rCBF and speech rate found in PD by introducing a left IFG subregion that increases with speech rate, but the conflicting left IFG subregion response remained. Conflicting IFG responses may account for some of the speech problems observed after STN-DBS. Cortical and subcortical regions may be differentially affected by STN-DBS.

## Introduction

High frequency, deep brain stimulation of the subthalamic nuclei (STN-DBS) has become a widespread tool in the treatment of levodopa responsive Parkinson's disease (PD), minimizing tremor and bradykinesia. Like levodopa, however, STN-DBS has had a less impressive impact on the axial symptoms, including speech. It is generally believed that STN-DBS can have an adverse effects on speech (1, 2) but the nature and extent of these changes have not been consistent and the pathophysiology is not known.

Speech studies with STN-DBS have typically evaluated one or more tasks including sustained vowel productions, syllable repetition, reading or repetition of text, or in some cases, spontaneously spoken monologs collected on or off medication, in subjects with varying degrees of Parkinsonian dysarthria. Small increases in the vocal intensity and fundamental frequency variability were observed during monolog speech but not vowel production with STN-DBS (3). The harmonic-to-noise ratio in conversational speech increased with STN-DBS to the normally higher level found during repeated speech (4). Inconsistent phonation effects during STN-DBS were found for males but not females (5). STN-DBS restricted articulatory space at the initiation of phonation (6), altered the pattern of pausing in spontaneous speech (7), increased production rates for words and clauses (8) and single syllables (9). Speech quality received lower ratings during STN-DBS (10).

The stimulating parameters in STN-DBS on speech have also been examined. At clinical settings, there were no significant STN-DBS on-off differences but intelligibility was reduced when comparing low frequency (70 Hz) to higher frequencies (130 and 185 Hz) (11) and comparing 4 to 2 v stimulation (12). The speech item on the Unified Parkinson's Disease Rating Scale (UPDRS) (13) suggested less impairment with low compared to higher frequency stimulation (14). Intelligibility tended to decrease with STN-DBS frequency, but pair-wise comparisons between individual frequencies were not significant (15). Speech during low frequency STN-DBS was judged to show increased weakness and instability. Higher frequency stimulation was associated with subjectively improved phonation and articulation (16).

Intelligibility and listener difficulty were evaluated from spontaneous speech with a frequency lower than the patient's clinical setting (low frequency stimulation—LFS) 60 Hz, the patient's clinical setting (high frequency stimulation—HFS) typically 185 Hz, and STN-DBS turned off (17). The use of spontaneous speech is significant, as the effects of STN-DBS on speech are more pronounced during spontaneous speech compared to reading or repetition (18–21). The results demonstrated that both LFS and HFS reduced intelligibility, 16 to 11%, respectively, compared to STN-DBS off. Simply reducing the frequency of STN-DBS stimulation did not improve the intelligibility of spontaneous speech as intelligibility was significantly higher with HFS compared to LFS. Difficulty ratings were not significantly different for LFS and HFS but on the average, intelligibility was inversely related to the difficulty ratings across STN-DBS conditions. Individuals who were hard to understand without STN-DBS were hard to understand with STN-DBS (22).

The STN-DBS effects on speech have been inconsistent (10) and the underlying causes are unknown. Positron Emission Tomography (PET) during a variety of speaking tasks has shown increased CBF on a whole-brain basis (23) and multi-focal increases using voxel-based principal component and linear discriminant analyses (24). The goal of the present study was to examine functional changes on a regional basis during speech production in individuals with PD, treated and untreated with STN-DBS. Positron Emission Tomography (PET) using a Performance-Based Analysis has produced a simple but reproducible, clinically relevant relationship between speech rate and rCBF in the left IFG region and the right CAU nucleus in normal (25, 26) and ataxic (27, 28) speakers.

## Methods and Materials

### Subjects

The demographic characteristics of the individuals in this study with idiopathic PD are presented in Table 1. There were seven PD subjects studied twice (age 63 ± 10 yrs) and seven PD STN-DBS subjects studied twice (on and off) (age 57 ± 5 yrs). All subjects were right handed. All subjects were native speakers of English, with the exception of one of the STN-DBS subjects. Although that subject self-referred as a native English speaker, he immigrated to the United States from Italy as an adolescent. All subjects abstained from their PD medications following the last evening dose the night before the study. The medical indications for STN-DBS were advanced, medically refractory PD with marked clinical swings between medication doses (i.e., on/off effects), as well as levodopa-induced dyskinesias. All STN-DBS was bilateral. STN-DBS amplitude varied across individuals for optimal therapeutic efficacy. STN-DBS on and off studies were performed on different days separated by at least 1 week. The order of the on and off scans was randomized, with four subjects being scanned off first and three subjects being scanned on first. For comparative purposes, the average ages of the non-PD normal control subjects (*n* = 16) was 57 ± 10 yrs (26) and the original normal group (*n* = 13) was 43 ± 11 yrs (27). The age of the hereditary ataxia group (*n* = 24) was 40.7 ± 17 yrs. While the ages of the present groups and the comparison groups span over three decades, the multiple-linear regression predictions of speech rates were replicable in the two normal groups and neurologically relevant in the cerebellar ataxia group.

**Table 1:** Demographic characteristics of the study subjects.

**Group**	**Age**	**Sex**	**Duration PD**	**Duration DBS**	**UPDRS-III**	**Levodopa**
PD	63 ± 10	4 (F) 3 (M)	10 ± 3	n.a.	20 ± 5	513 ± 300
DBS	57 ± 5	7 (M)	12 ± 3	26 ± 21	35 ± 18 (off) 26 ± 16 (on)	450 ± 150

*Age and duration of DBS are in years. Duration of DBS is in months. Sex: M, male; F, female. UPDRS-III is the Unified Parkinson's Disease Rating Scale, part III (motor examination)*.

#### Sample Size

The sample sizes of the study were determined by several factors: One perspective includes finite financial and technical resources to utilize PET scanning to accomplish STN-DBS on and off studies along with matched PD and normal control participants. The other factor was the ability to recruit (1) PD subjects willing to forego their PD medication, and (2) PD STN-DBS participants who agree to forego both PD medication and STN-DBS stimulation; these study requirements constitute challenges for persons with PD. As there were no previous comparable PET studies involving speech in STN-DBS, we felt that the examination of the results from PB, STN-DBS ON, STN-DBS OFF, and healthy control speakers could provide a broader context to better interpret the effects of STN-DBS. Our previous studies suggested that the current sample size would be adequate especially since we optimized the scanning protocols by replacing “resting scans” with additional speech conditions. Further, our previous studies have yielded comparable and replicable brain-speech relationships in neurological and healthy subjects whose ages spanning over three decades suggesting that the age differences in the participants in this study would not be a factor.

### Positron Emission Tomography Imaging Procedures

Participants typically arrived at the PET suite at 8:00 AM to be consented, interviewed, and briefed in the scanning procedures. Participants were positioned in the PET scanner (GE Advance Tomograph, General Electric) (29) and an intravenous line was placed in the subject's left arm for ${\text{H}}_{2}^{15}$O injection, which occurred at ~10:00 am. Stable and reproducible head positioning was accomplished with a stereotactic head-holder and 3D laser alignment. Communication with the subject was facilitated with lightweight headphones attached to the head-holder. A transmission scan (10 min) was performed for attenuation correction followed by a 2D PET scan to establish the delay time between ${\text{H}}_{2}^{15}$O injection and the detection of brain activity by the scanner. A series of whole-brain 3D PET scans followed, with two scans for each of the three speech repetition tasks. Based on the observed brain delay time, each speech task was initiated 15 s prior to detection of ${\text{H}}_{2}^{15}$O in the brain. Tasks were performed for 60 s using the procedure reported previously (25–28, 30). A modified slow bolus injection of ${\text{H}}_{2}^{15}$O using an automated injection system was used to measure blood flow. Image acquisition lasted approximately 2 min.

### PET Image Processing

Scans were reconstructed using 3D reprojection (3D RP) method, matrix dimensions 128 × 128 × 35, with voxel dimensions of 2.34 × 2.34 × 4.25 mm, with no smoothing applied. PET images were first aligned within subject and then spatially normalized to a standard space using the SPM99 software (31). The normalized voxels have the dimension of 2 mm in all directions. Regions of interest used in previous PET-speech studies (25–28, 30) were used as the basis for extracted multiple regional CBF values from eight axial slices from the ventral to dorsal extent of the head of the CAU, and regional values from eight axial slices, the ventral to dorsal extent of the IFG regions, bilaterally. Data were extracted using ScanVP image analysis software (32). Irregular regions were used and adjusted on an individual basis to ensure capture of the target structure. However, regions were constant within a subject across all speech conditions. A threshold was applied to each region so that the upper 10% of activity was captured to reduce partial volume errors and to minimize individual differences in anatomy. For each scan, a global CBF value was obtained using a whole-brain region of interest. This was used for normalization across subjects.

### Speech Samples

Three speech tasks were used: repeated productions of the following: syllable /*pa*/, the syllable sequence /pa-ta-ka/, and the sentence /pop-the-top-cop/. Each task was performed twice, each occurrence lasted 60 s, and each was associated with a PET scan. These tasks were performed in random order in the first half of the study and then repeated in reverse order in the second half. The speech samples used to extract dependent measures for the performance-based analysis were digitally recorded during scanning. Syllable rates were measured. Syllable rate refers to the number of syllables per second produced by each speaker during the 60 s production period.

### Statistical Analysis

**Figure 1:**
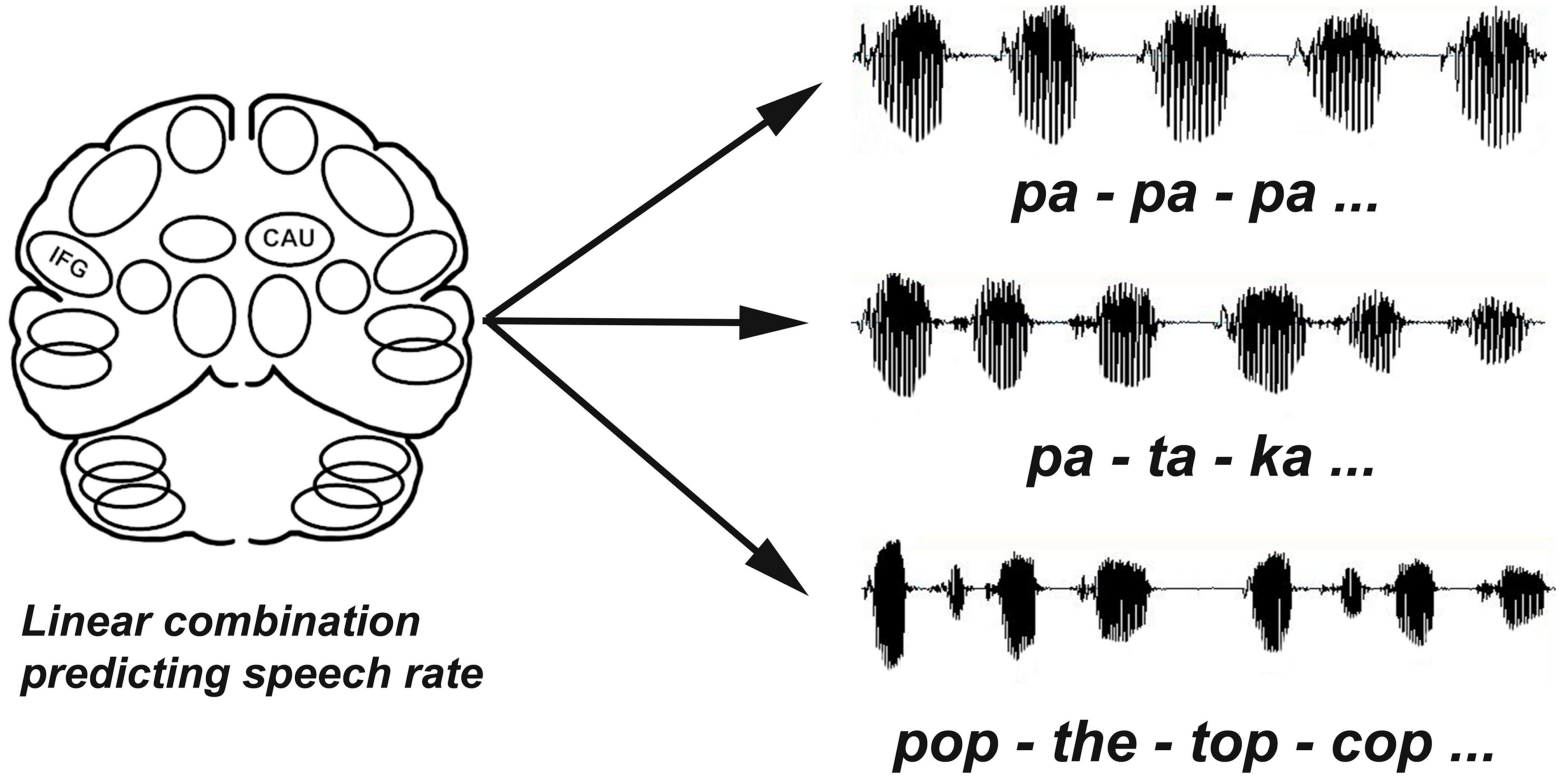
Repetition rates (syllables/second) were calculated for each task (/*pa-pa-pa, pa-ta-ka, pop-the-top-cop*). The performance based analysis used multiple linear regression to evaluate blood flow data for multiple slices through the left and right IFG and CAU regions as independent variables to determine if there was a combination of regions that significantly predicted speech rates (dependent variable) across tasks.

For the Performance-Based Analysis of speech rate, the rCBF data were normalized using the ratio between the highest whole-brain rCBF value in the dataset and the whole-brain CBF value for the scan from which the regional values were measured (25–28, 30, 33). These globally normalized rCBF data from the left and right heads of the CAU nuclei and IFG regions for each of the repetition task scans were used as predictor variables for the repetition rate measured during each scan (outcome variable) in a stepwise multiple linear regression analysis (34). The Performance-Based Analysis uses the stepwise multiple linear regression to determine if there is a linear combination of regional rCBF data that predicts a performance measure such as repetition rate or vocal stability (33, 34). This statistical procedure assesses the contribution of each potential predictive region to establishing a significant linear relationship with the dependent variable (Figure 1). Regions are entered into a regression model, tested, and either retained or rejected. The following criteria were used for all regression analyses: probability of *F* to enter (0.05), probability of *F* to remove (0.10), and tolerance (0.01). While over-fitting and under-fitting regression models can be a concern with this approach, cross-validation is recommended as a confirmatory procedure. The prediction of speech rate provided a cross-validation of the stepwise multiple regression analysis with previous functional imaging studies by replicating their results (25–28, 34). Moreover, the brain regions identified using the stepwise multiple liner regression replicated the effects of brain lesions to these areas in clinical studies, supporting the validity of the analysis.

In addition to the Performance-Based Analysis, dependent and independent groups *t*-tests and Spearman (non-parametric) correlations were used to examine the dataset (35). Neither the image analyses nor the speech analyses were conducted in a blinded fashion.

## Results

Repetition rates (syllables/second) across the three tasks were greater for the PD group (mean ± standard deviation = 3.8 ± 0.8) compared to the STN-DBS group in the off (2.9 ± 1.1) [*t*_(124)_ = 5.2; *p* < 0.0001] and on conditions (3.0 ±1.0) [*t*_(124)_ = 4.6; *p* < 0.0001]. Repetition rates did not differ for the STN-DBS off and on conditions. For comparison, repetition rates for the age-matched normal reference group was 4.1 ± 0.8.

### Subject Factors Influencing Speech Rate

#### Parkinson's Group

Repetition rates were not correlated with age, the duration of PD, UPDRS motor score, or the daily dose of Levodopa.

#### STN-DBS ON

Repetition rates decreased as the amplitude of the left electrode increased (*r* = −0.52; *p* < 0.001). The amplitude of the right electrode was not associated with repetition rate. Repetition rates decreased as the duration of STN- increased (*r* = −0.63; *p* < 0.001) and the UPDRS-III increased (*r* = − 0.61; *p* < 0.001).

#### STN-DBS OFF

Repetition rates decreased as the duration of STN-DBS increased (*r* = −0.67; *p* < 0.001). Repetition rates also decreased as the duration of PD (*r* = −0.4; *p* < 0.01) and the UPDRS-III scores increased (*r* = −0.53; *p* < 0.001). Repetition rate also increased with the daily dose of Levodopa (*r* = 0.41; *p* < 0.01).

### Performance Based Analysis

#### Parkinson's Group

**Figure 2:**
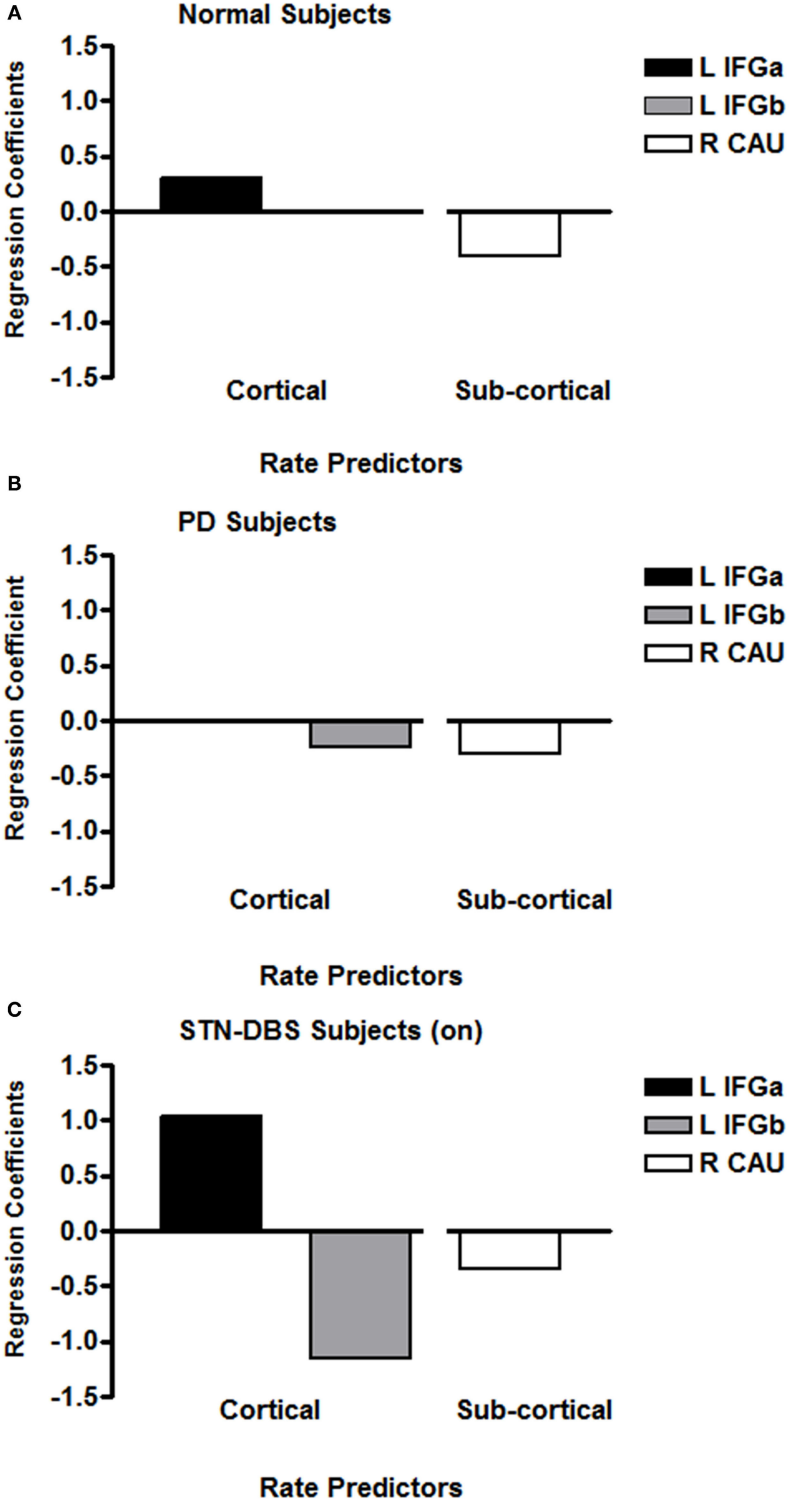
The regression coefficients for significant multiple linear regression models that predicted syllable repetition rates for previously published age-matched normal subjects **(A)**, individuals with PD **(B)**, and individuals with PD treated with STN-DBS **(C)**. The axial slices through the left IFG are at the lowest two levels (within 4 mm), with the normal IFG predictor superior and adjacent to the PD IFG. The left IFG predictor subregion (L IFGa) that increased with speech rate with STN-DBS on is at the same axial level as the left IFG predictor subregion (L IFGa) that increased with speech rate in the normal group. The left IFG predictor subregion (L IFGb) that decreased with speech rate with STN-DBS on was at the same axial level as the left IFG predictor subregion (L IFGb) that decreased with speech rate in the PD group. The normal right CAU predictor is at the lowest axial slice but the PD CAU predictor is at a level that is 14 mm superior to the normal predictor. The right CAU predictor region that decreased with speech rate with STN-DBS on was at the same level as the right CAU region that decreased with speech rate in the PD group.

A stepwise multiple linear regression included speech rate as the dependent variable and the left-right pairs of IFG and CAU regions across eight 2 mm transverse axial slices plus the daily dose of levodopa as independent variables. A significant predictive pattern was observed for speech rate for the phonological and lexical repetition tasks [*F*_(2,81)_ = 6.87; *p* = 0.002]. The model consisted of a negative standardized beta regression weight (−0.29) for a right CAU region as well as a negative standardized beta regression weight (−0.24) for a left IFG. The Levodopa dose did not contribute to the predictive model. Figure 2A presents the previously published results for normal subjects (24) for comparison with the results obtained for the PD group (Figure 2B). The negative weight for the PD left IFG is at odds with previous results obtained for two normal groups (25, 26) and for three genotypes of spinocerebellar ataxia (27, 28).

#### STN-DBS OFF

A third parallel stepwise multiple linear regression was applied to the data obtained with the STN-DBS turned off. A significant predictive pattern for speech rate for the repetition tasks was obtained for a single region [*F*_(1,40)_ = 15.99; *p* < 0.001]. This model consisted of a negative standardized beta coefficient (−0.53) for a left CAU region. The daily dose of Levodopa did not contribute to the model.

#### STN-DBS ON

A stepwise multiple linear regression parallel to that used for the PD data was applied to the data obtained from the STN-DBS data with the stimulation on. A significant predictive pattern for speech rate for the repetition tasks was obtained [*F*_(4,37)_ = 10.08; *p* < 0.001]. This model consisted of a negative standardized beta coefficient (−0.34) for the same right CAU region identified in the PD model, a negative standardized beta coefficient (−1.15) for the same left IFG identified in the PD model, and a positive standardized beta coefficient (+1.15) for the left IFG region immediately superior to the negatively weighted IFG region. Although the subjects were scanned off medication, the daily dose of levodopa also contributed to the model (+0.51), probably as a marker of PD severity. The predictor model coefficients for the STN-DBS on condition are presented in Figure 2C.

## Discussion

Speech in PD has been characterized in many ways, but deficiencies in elements of motor control (e.g., volume, rate, articulation, and prosody) rather than motor coordination are important features of its impairment. A combination of rCBF in the left IFG and right CAU has been associated with speech rate, but in PD, unlike in normal subjects and individuals with spinocerebellar ataxia, the left IFG contribution decreased rather than increased with increasing speech rate. Decreased rCBF coefficient activity in the left IFG during speech production is consistent with poor motor control for speech in PD.

The results in the individuals with STN-DBS turned off somewhat surprisingly did not mimic the results obtained from the PD individuals not treated with STN-DBS. The left CAU rCBF activity observed during speech with STN-DBS off does not fit any previously observed pattern. There are several possibilities for this. Although the subjects treated with STN-DBS were slightly younger that the PD subjects, the cortical-subcortical pattern associated with speech rate has been observed across an age range of over three decades. The effects of surgery may also play a role, but the abnormal PD pattern is seen in the same individuals when the STN-DBS was turned on. Much is not known about the long-term effects of STN-DBS. Most likely the left CAU effect reflects functional reorganization following an average of 26 months of continuous STN-DBS, an uninterpretable pattern would occur after a 2 h period without stimulation.

In the STN-DBS subjects with the stimulators tuned on, the abnormal left IFG coefficient observed in the PD group was also found. In addition, a left IFG coefficient that increased with speech rate was found at an anatomic level superior to the abnormally decreasing level. This pattern consisted of the normal and ataxic cortical-subcortical relationship as well as the abnormal left IFG decreased coefficient in the PD group. The STN-DBS introduced some normalization of the cortical-subcortical pattern during speech, but it did not eliminate the abnormal left IFG activity that was present in the PD group. The conflicting left IFG responses during STN-DBS may well-account for the difficulties in speaking reported by some individuals.

The STN appears to play a role in speech initiation and pausing. Recordings of STN activity during reading obtained during implantation surgery demonstrated increased firing rate synchronized to the onset of speech (36). Local field potentials recorded during electrode placement in the STN revealed high-gamma power specific to the articulator (tongue, lips) starting with the onset of articulation and continued for its duration (37). At the initiation of sustained phonation, vowel articulation is reduced when STN-DBS is on but not when it is off (6). In spontaneous speech, abnormal pausing occurs with STN-DBS with significantly shorter long pauses that were more likely to occur in non-linguistic boundaries (7).

A notable aspect of the left IFG rCBF coefficients during STN-DBS is that both responses are exaggerated when compared to what has been observed in normal, ataxic, and PD speakers. These exaggerated responses may be related to the global and multi-focal increases in CBF following STN-DBS (23, 24). However, the observation that the right CAU response was not exaggerated with STN-DBS suggests that this effect is not uniform (24). For speech, STN-DBS appears to have a greater effect in modulating the cortical functional responses compared to the subcortical responses.

Consistent with role of the left IFG in the predictive model of speech rate was the observation that speech rate decreased as the amplitude of the left electrode increased but the amplitude of the right electrode was not associated with speech rate. It has also been reported that subjective ratings of prosody, articulation, and intelligibility declined during left vs. right STN-DBS (38). Similarly, vowel production was observed to be more adversely affected by left compared to right stimulation (39).

With respect to the rCBF activity observed during speech with STN-DBS off, the left CAU activity does not fit any previously observed pattern. While the symmetry of experimental design suggests that STN-DBS on and off condition be compared, it should not be a surprise that following an average of 26 months of continuous STN-DBS, an uninterpretable pattern would occur after a 2 h period without stimulation.

The cortical-subcortical rCBF patterns reported in this paper represent state-like, acute rCBF changes identified by virtue of their relationships with actual symptomatic behaviors engaged in during scanning (40). This approach contrasts with more trait-like, chronic patterns of metabolic abnormality that better represent the presence of disease (41–43), its progression and response to treatment (44–46), its components (47), and variants (48, 49). The state-like approach has advantages for understanding functional neuropathology underlying specific neurologic signs (e.g., Parkinsonian speech) whereas the trait-like approach represents a more stable, chronic condition that is advantageous in establishing diagnoses and severity. It is likely that a specific brain stimulation has different effects on state-like and trait-like networks.

A simple model of cortical-subcortical interaction has been shown to reliably be associated with speaking rate in normal, ataxic, and PD. This elementary network provides a starting point for examining the undoubtedly more complex neural activity underlying speech as reflected in rCBF. However, the predictive pattern of rCBF activity represents the function of populations of neurovascular units (NVUs) within each region of interest and not simply neuronal activity. The NVU consists of neurons, glia, and blood vessels (50). Its activity reflects the influences of neurotransmitters and other vasoactive mediators on each of these cell types as well as metabolic demand (51, 52). In a mouse model, Han et al. (53) demonstrated that stress could disrupt the neuronal excitatory-inhibitory relationship changing the relationship between neuronal activity and the associated vascular response (neurovascular coupling). While STN-DBS might affect neurovascular coupling on a global or multi-regional basis during speech, neuronal coupling between blood flow and metabolism has been shown to be affected by levodopa, especially in individuals with levodopa induced dyskinesia, but not STN-DBS during the rest state (54). On a regional basis, however, coupling may be different during behavioral stimulation (55).

The interactions among the characteristics of brain networks, therapies, and behavior is undoubtedly complex. In the present study, the IFG rCBF predictors of speech rate were exaggerated during STN-DBS without a significant change in speech repetition rates. In contrast, STN-DBS has been shown to produce a spatial rCBF pattern associated with improved visual-motor learning task learning but levodopa infusion did not (56). Regional cerebral blood flow is a useful surrogate marker of neurological systems, but there is no direct correspondence between rCBF markers and specific behaviors. Rather, the changes between rCBF networks and performance during STN-DBS represent changes in the metrics that connect brain activity and behavior. This is an example of the complexity entailed in interpreting the actual rather than inferred relationships between surrogate markers of brain function and tangible performance.

In summary, PD patients not treated with STN-DBS demonstrated an abnormal pattern of rCBF during speech repetition: as in normal and ataxic speakers, rCBF predictive coefficient decreased in the right CAU as speech rate increased; unlike the normal and ataxic speakers, the rCBF predictive coefficient in the left IFG contribution also decreased. This may reflect a significant factor in the abnormal motor control of speech in PD. During STN-DBS, the cortical-subcortical rCBF pattern was normalized somewhat by introducing a left IFG predictive coefficient in which the rCBF contribution increased with speech rate. However, the left IFG region in which the contribution decreased with speech rate identified in PD remained. Conflicting IFG subregions with exaggerated increasing and decreasing rCBF contributions may account for the inconsistencies in the speech difficulties reported following STN-DBS. This therapy also appears to alter the metrics that characterize the relationship between rCBF and behavior.

## Data Availability Statement

The raw data supporting the conclusions of this article will be made available by the authors, without undue reservation.

## Ethics Statement

All subjects provided informed consent for the speech (The Nathan Kline Institute/Rockland Psychiatric Center Institutional Review Board; Institutional Review Board of the Mount Sinai School of Medicine) and PET (Feinstein Research Institute, Northwell Health Institutional Review Board) components of this study in accordance with the Helsinki Declaration of 1975 (and as revised in 1983). The patients/participants provided their written informed consent to participate in this study.

## Author Contributions

JS and DS designed the study and evaluated the speech data. MT characterized the subjects. VD, DE, and JS conducted the PET imaging. JS conducted the analysis. All authors contributed to the article and approved the submitted version.

## Conflict of Interest

The authors declare that the research was conducted in the absence of any commercial or financial relationships that could be construed as a potential conflict of interest.
